# Optimized Golgi-Cox Staining Validated in the Hippocampus of Spared Nerve Injury Mouse Model

**DOI:** 10.3389/fnana.2020.585513

**Published:** 2020-11-09

**Authors:** Jia-wei Zhang, Sidra Tabassum, Jin-xiang Jiang, Cheng Long

**Affiliations:** ^1^Panyu Central Hospital, South China Normal University-Panyu Central Hospital Joint Laboratory of Translational Medical Research, Guangzhou, China; ^2^Precise Genome Engineering Center, School of Life Sciences, Guangzhou University, Guangzhou, China; ^3^School of Life Sciences, South China Normal University, Guangzhou, China

**Keywords:** modified Golgi-Cox staining, neuronal morphology, spared nerve injury, mouse model, hippocampus

## Abstract

Golgi-Cox staining has been used extensively in neuroscience. Despite its unique ability to identify neuronal interconnections and neural processes, its lack of consistency and time-consuming nature reduces its appeal to researchers. Here, using a spared nerve injury (SNI) mouse model and control mice, we present a modified Golgi-Cox staining protocol that can stain mouse hippocampal neurons within 8 days. In this improved procedure, the mouse brain was fixed with 4% paraformaldehyde and then stored in a modified Golgi-Cox solution at 37 ± 2°C. The impregnation period was reduced from 5–14 days to 36–48 h. Brain slices prepared in this way could be preserved long-term at –80°C for up to 8 weeks. In addition to minimizing frequently encountered problems and reducing the time required to conduct the method, our modified protocol maintained, and even improved, the quality of traditional Golgi-Cox staining as applied to hippocampal neuronal morphology in SNI mice.

## Introduction

Golgi staining, pioneered by Camillo Golgi in 1873 (Altamura, 1996; Dal Canton et al., 1999), has a long history of applications in neuronal and glial morphology (Simons and Woolsey, 1984; Ranjan and Mallick, 2012). For example, it has been widely used to investigate changes in neuronal development (Singh, 1980; Buell, 1982), and to study pathology (Simons and Woolsey, 1984; Yoshioka et al., 1986; Faherty et al., 2003; Marín-Padilla et al., 2003) and death (Simons and Woolsey, 1984; Gibb and Kolb, 1998; Zaqout and Kaindl, 2016) of neural cells. The classical Golgi method was modified by Cajal and has been referred to as the rapid Golgi staining method (Cajal, 1891). Cajal used this method extensively to demonstrate previously unimagined neuronal morphology throughout the nervous system. He was the first to describe spines as small thorns protruding from the dendrites of cerebellar Purkinje neurons (García-López et al., 2007; Yuste, 2015). Today, dendritic spines are known to be centers of information processing with the ability to regulate their own protein synthesis and degradation (Halpain et al., 2005).

Golgi staining involves impregnation of neurons with metals and can stain the whole neuron, including the soma (Simons and Woolsey, 1984; Patrick and Anderson, 1995; Gibb and Kolb, 1998), axon (Faherty et al., 2003; Marín-Padilla et al., 2003), and dendrites (Zhang et al., 2011; Koyama et al., 2013; Levine et al., 2013). It is one of the most popular methods for studying neuronal morphology because of its convenience, low cost, an excellent display of detail, and a requirement for relatively simple microscopes alone (Scheibel and Tomiyasu, 1978; Gibb and Kolb, 1998; Faherty et al., 2003). Most researchers today rely on a two-step Golgi-Cox staining procedure (Zhong et al., 2019); chromation (potassium chromate and potassium dichromate solution) followed by silver nitrate impregnation that subsequently allows the formation of silver chromate crystals, visualized as brownish-black structures (Zhang et al., 2010). However, it is unpredictable and time-consuming nature has resulted in several modifications of the protocol involving the composition and pH of the impregnation solution (Bertram and Ihrig, 1957; Morest and Morest, 1966; Adams, 1979; González-Burgos et al., 1992), the use of single sections for staining (Landas and Phillips, 1982; Gabbott and Somogyi, 1984), the use of microwaves (Armstrong and Parker, 1986; Berbel, 1986), changes in the embedding media (Blackstad et al., 1984; Kolodziejczyk et al., 1990), the use of a vibratome (Patro et al., 2009), coating of brain blocks with egg yolk (Zhang et al., 2010), application of vacuum (Friedland et al., 2006), auto-metallographic enhancement (Orlowski and Bjarkam, 2009), and variation in temperature of the tissue-incubation media (Ranjan and Mallick, 2010; Narayanan et al., 2014), among others. Most modifications have aimed to decrease the time required for the procedure while maintaining the quality of the staining. However, the modified protocols reported are still time-consuming with insufficient clarity of the neuronal morphology (Du, 2019; Sivaguru et al., 2019). Aside from this, the long fixation period turns the tissue brittle, making sectioning more difficult. Moreover, strongly stained blood vessels appear as background, thereby interfering with the interpretation of the neuronal structures.

Here, we report a simpler, faster, and more efficient Golgi-Cox staining protocol for reliable, high-quality staining in an acceptable time frame and with well-preserved tissue quality. Using this modified method, we validated the effects of different fixation and impregnation times and defined suitable temperatures for the long-term preservation of brain slices. We also compared the modified protocol with the traditional method on hippocampal neurons of spared nerve injury (SNI) model and control mice.

## Materials and Methods

### Animals

Twelve male C57BL/6 mice were provided by Guangdong Medical Laboratory Animal Center were used in this study. Mice were housed in a climate-controlled room (25°C) under a 12-h light/dark cycle (lights on at 8:00 a.m.). Food and water were supplied *ad libitum*. All experiments and animal housing were conducted following procedures approved by the Ethics Committee for animal research at South China Normal University, consistent with the guidelines for the care and use of laboratory animals established by the National Institutes of Health.

### Surgical Procedures

To establish the SNI mouse model, mice were anesthetized by intraperitoneal injection (i.p.) of 0.4 g/kg chloral hydrate (Sigma, USA). The common peroneal and tibial nerves of mice were explored, tightly ligated with silk, and transected distal to the ligation, removing a 2–4 mm length of each nerve. Care was taken to avoid any contact with or stretching of the intact sural nerve, leaving the remaining sural nerve intact (Decosterd and Woolf, 2000; Kobiela Ketz et al., 2017; Cichon et al., 2018). On day 7 after the operation, the mouse brain was taken for Golgi-Cox staining.

### Modified Golgi-Cox Staining

**Figure 1:**
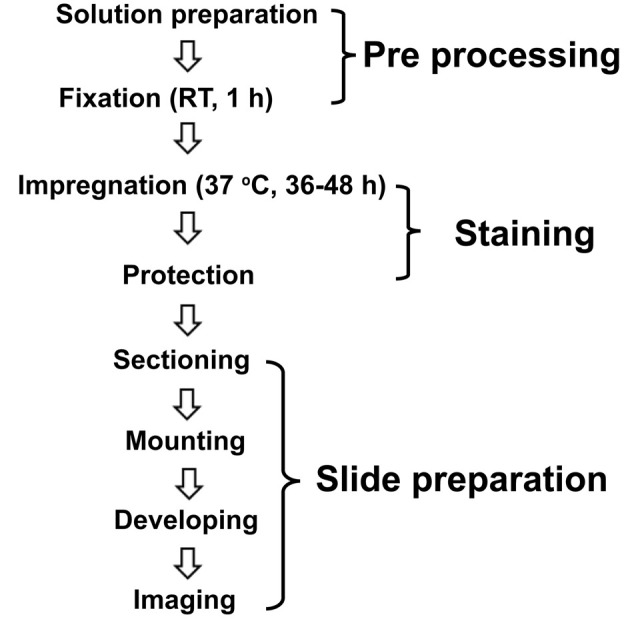
Schematic illustration of the modified Golgi-Cox staining protocol used in this study. The optimized protocol consists of three major steps of pre-processing, staining, and slide preparation. For pre-processing, the brains incubated in fixative (4% PFA) for 1 h at room temperature (RT) right after the perfusion. Following fixation, the brains incubated in the Golgi-Cox staining solution for 48 h, at 37°C, and then in protection solution at 4°C followed by slide preparation step.

We performed a modified protocol of Golgi-Cox staining based on previously described protocols (Ranjan and Mallick, 2010; Zaqout and Kaindl, 2016) and optimized several methodological steps, as described in Figure 1. The compounds were purchased from Guangzhou Chemical Reagent Factory (China) except where mentioned.

#### Preparation of Golgi-Cox Solution

The modified Golgi-Cox staining solution was prepared from three different solutions as follows: solution # 1: 5% potassium dichromate (K_2_Cr_2_O_7_) solution [5 g K_2_Cr_2_O_7_ dissolved in 100 ml double-distilled water (ddH_2_O)]; solution # 2: 5% mercuric chloride (HgCl_2_) solution (5 g HgCl_2_ dissolved in 100 ml ddH_2_O); and solution # 3: potassium chromate (K_2_CrO_4_) solution (4 g K_2_CrO_4_ dissolved in 80 ml ddH_2_O and topped up with 200 ml ddH_2_O).

The whole volumes of solutions # 1 (100 ml) and # 2 (100 ml) were thoroughly mixed and then added to solution # 3 (280 ml), resulting in a total of 480 ml modified Golgi-Cox staining solution. The modified Golgi-Cox staining solution was stored in the dark at room temperature (RT) for 1 day; it was then filtered to avoid the formation of red/yellow precipitate (mercuric chromate) on the brain sections.

#### Perfusion and Fixation

Intracardial perfusion was performed with 200 ml of 0.9% saline at RT in mice anesthetized by chloral hydrate (Sigma, USA; 0.4 g/kg, i.p.). Right after perfusion, the brains were fixed in 10 ml bottles filled with 5 ml 4% paraformaldehyde (PFA). To investigate the effect of fixation time on the staining quality of the tissue, brains were fixed in 4% PFA for 10, 40, or 70 min.

#### Impregnation

Following fixation, both SNI and control brains were transferred to 50 ml brown bottles filled with 10 ml modified Golgi-Cox solution. As mentioned earlier, the optimal impregnation temperature is 37°C (Ranjan and Mallick, 2010); hence we incubated a total of eight mice brains at 37°C in the dark for 24, 36, 48, 72, or 144 h in pairs. Previously Ranjan and Mallick (2010) used tissue block for incubation in the Golgi Cox staining solution. They mentioned that 24 h incubation in the Golgi Cox solution is optimal to stain tissue blocks. Moreover, in another study, Ranjan and Mallick (2012) described brain incubation time in the Golgi Cox staining solution for 48 h. Therefore, we have incubated the whole brain in the Golgi Cox staining solution, for 24, 36, 48, 72, or 144 h for optimal impregnation time. The solution was changed every 24 h.

#### Tissue Protection

To protect brain tissue, brains were placed in small bottles in protecting solution (300 g/l sucrose in ddH_2_O) and transferred to 4°C in the dark. The protecting solution was changed every day until the solution became clear (4–6 days).

#### Tissue Sectioning

Brains were taken out of the storage solution, dried on filter paper, and cut into 80–200 μm sections with a cryostat (Leica, Germany). Sections were kept in a 30% sucrose solution in phosphate-buffered saline (PBS, 0.01 M, pH = 7.4) before being transferred and unfolded onto microscope glass slides. The absorbent paper was used to remove the excess sucrose solution.

#### Tissue Mounting

Microscope slides (Citotest, China) coated with 1.5–2% gelatin were used in this study. The slides were transferred to a glass staining vial (Citotest) for color development and then left to dry at RT until the brain slices turned pale yellow. To observe the effects of different temperatures on long-term preservation of brain slices, tissue sections were protected with sealing film (Citotest) and subjected to gradient freezing at 4, −20, or −80°C for up to 8 weeks. Because direct storage of brain slices at −80°C could have resulted in the fragmentation of brain slices, for long term storage of brain slices in −80°C, the slides were first kept in 4°C and −20°C for 30 min each, then stored at −80°C.

#### Tissue Developing

Brain slices were washed with ddH_2_O and 50% ethanol for 5 min. They were then rinsed with 25% ammonia solution and placed in the dark for 30 min. Next, slices were washed again with ddH_2_O for 5 min and then kept in 5% sodium thiosulfate in the dark for 30 min. Finally, slices were washed with 70%, 90%, and 100% ethanol for 6 min each, and immersed in xylene for 6 min to reduce the background.

#### Imaging

Images were captured using a microscope (EVOS FL Auto; Thermo, USA) at both low (10×) and high (20×) magnifications. Representative images from the hippocampus were taken from 5 to 7 cells per brain slice. To examine whether various parameters of stained neurons could be quantified, two parameters (total dendritic length and neural spine density) were analyzed using ImageJ (NIH, USA). Neural spine density, the number of dendrites, and dendritic intersections were analyzed by the concentric circle method reported by Sholl (Sholl, 1953; Zahra et al., 2018). The number of branches and intersections were counted up to a distance of 140 μm from the soma. By measuring two parameters, tracing and quantification could be performed in both groups in a comparable way. The length and branches of the dendrites, along with the neuron intersections, were marked with concentric circles spaced at a distance of 10 μm. The dendrite density depended on the number of branch bifurcations (Sholl, 1953; Graveland et al., 1985; Friedland et al., 2006; Koyama and Tohyama, 2013).

### Statistical Analysis

All data were expressed as the mean ± SEM. Origin 8.0 software (OriginLab, USA) was employed for statistical analysis and generating graphs. Statistical analyses were performed using one-way analysis of variance (ANOVA). For the intersection of multiple groups, data were analyzed using two-way repeated-measures ANOVA with Tukey’s *post hoc* test. *p* < 0.05, *p* < 0.01 and *p* < 0.001 were accepted as statistically significant and denoted *,** (## or $$) and ***, respectively.

## Results

### Optimization of the Golgi-Cox Staining Conditions

#### Fixation Time

**Figure 2:**
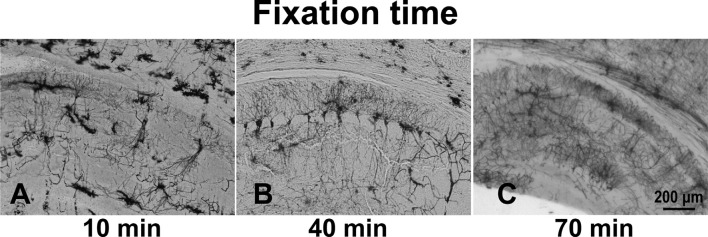
The effect of immersion of brain tissue in 4% PFA for different times: 10 min **(A)**, 40 min **(B)**, and 70 min **(C)**.

The optimal fixation time was determined by fixing brains in 4% PFA for 10, 40, or 70 min. The 10-min fixation of the brains resulted in a high background (Figure 2A). Brain tissue immersed in fixative for 40 min (Figure 2B) and 70 min (Figure 2C), however, showed better staining results. Specifically, all stained hippocampal neurons, including the dendritic spines, exhibited an almost entirely black stain, resulting in sharp images showing complete cell structure.

#### Impregnation Period

**Figure 3:**
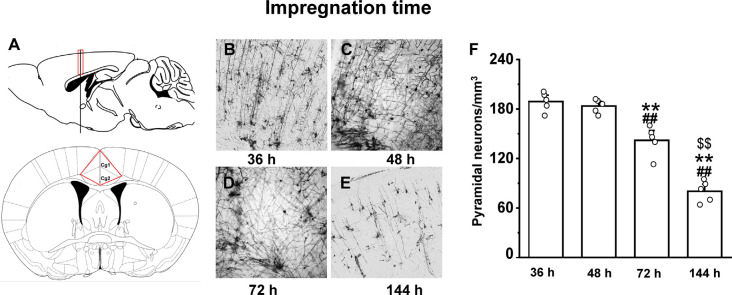
The impact of impregnation time on brain tissues in an optimized Golgi-Cox staining solution. Schematic diagram showing the anterior cingulate cortex region **(A)**. Golgi-Cox staining results after 36 h **(B)**, 48 h **(C)**, 72 h **(D)**, and 144 h **(E)**. The density of pyramidal neurons in the cortex at different impregnation times **(F)**. **Indicate the significant difference of other impregnation times with 48 h, ^##^indicates the significant difference of other impregnation times with 36 h, and ^$$^indicate the significant difference of other impregnation times with 72 h. Each value represents mean ± SEM; **^,##,$$^*p* < 0.01.

The optimal incubation time in the Golgi-Cox staining solution was determined by testing 24, 36, 48, 72, and 144 h periods. In the 36 h incubation, although we observed an almost equal number of neurons in the anterior cingulate cortex region (Figure 3A) as of 48 h of impregnation time (*p* = 0.9), the quality of staining was very low throughout the brain (Figure 3B). The brain tissues exposed to the Golgi-Cox solution for 48 h (Figure 3C) at 37°C resulted in detailed dendritic trees and a better staining effect on pyramidal neurons than that produced by the 72 h incubation time (*p* < 0.01; Figure 3D). However, 144 h incubation periods resulted in the disappearance of many neurons (*p* < 0.01; Figure 3E): numerous black spots appeared in the background, and a few neurons—mostly randomly distributed in the hippocampal regions of the sections—were incompletely stained, resulting in only the backbone being visible. Figure 3F shows that the density of pyramidal neurons is relatively changed at different impregnation times, ranging from the highest at 36 h and 48 h, and lowest at 144 h (*F*_(3,19)_ = 73.602). Also, a fewer number of neurons and quality of staining was observed in the 24 h incubation compared to that in the 36 h incubation (Supplementary Figure 1).

#### Temperature for Long-Term Tissue Preservation

**Figure 4:**
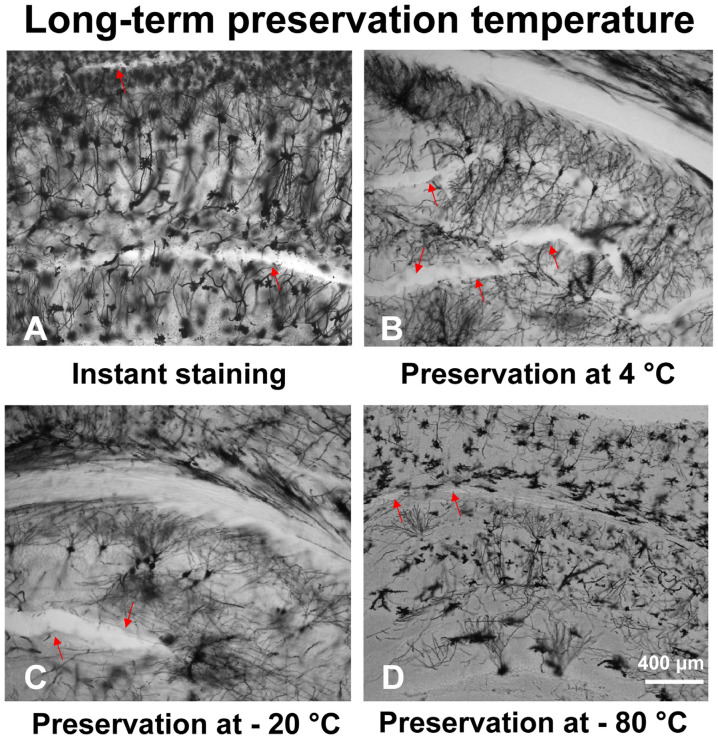
The effect of different temperatures on the long-term preservation of brain slices. The brain slices stained immediately after sectioning **(A)**, brain slices stored at 4°C **(B)**, −20°C **(C)**, and −80°C **(D)**.

With our modified method, brain slices can be preserved long-term (up to 8 weeks) at low temperatures to prevent tissue fragmentation (Figure 4D). The temperature of the brain slices was dropped step by step after mounting. First, the brain slices were stored at 4°C for 30 min, then stored at −20°C for 30 min. After that, the brain slices were stored at −80°C for up to 8 weeks. Arrow marks in the figures represent fragmentation location. Our results describe that brain slices stained immediately after sectioning (Figure 4A) and stained after long-term storage at −80°C (Figure 4D), showed less fragmentation. However, the brain slices stored at 4°C (Figure 4B) or −20°C (Figure 4C) showed multiple fragmentation locations.

### Comparison Between Traditional and Modified Golgi-Cox Staining Protocols

**Figure 5:**
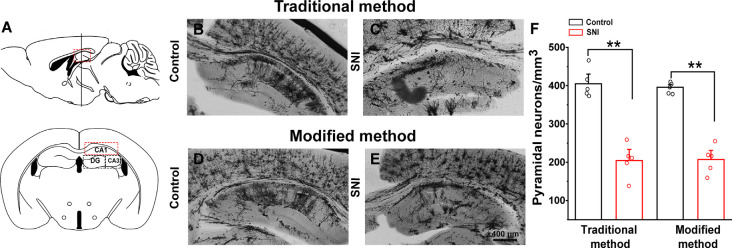
Comparison of traditional and modified Golgi-Cox staining methods on hippocampal neurons of control and spared nerve injury (SNI) mice. Schematic diagram of the hippocampal region **(A)**. Hippocampal pyramidal neurons of control mice with traditional Golgi-Cox staining **(B)**. Hippocampal pyramidal neurons of SNI mice with traditional Golgi-Cox staining **(C)**. Hippocampal pyramidal neurons of control mice with the modified Golgi-Cox staining protocol **(D)**. Hippocampal pyramidal neurons of SNI mice stained with the modified Golgi-Cox staining protocol **(E)**. The density of pyramidal neurons in the hippocampus CA1 regions of control and SNI mice using traditional and modified Golgi-Cox staining method **(F)**. Each value represents mean ± SEM; **p < 0.01.

We examined the hippocampal neuronal morphology of the control and SNI mice and compared the traditional (26 days) and our modified (8 days) Golgi-Cox staining methods. SNI mice displayed fewer hippocampal pyramidal neurons (Figures 5A,C) than the controls (Figures 5B,D) using the traditional method. Dendritic length and spine density were also significantly reduced in SNI mice. Similar results were obtained using the modified Golgi-Cox staining method (Figures 5C,D). The density of pyramidal neurons (Figure 5E) stained by traditional and modified method showed reduced number of neurons in hippocampal CA1 regions of SNI mice compared to control (*F*_(1,9)_ = 60.650, *p* < 0.01) and (*F*_(1,9)_ = 113.884, *p* < 0.01), respectively. These results speculate that the modified protocol is accurate and reliable, and provide increased reliability, and reproducibility of staining neurons.

### Validation of the Modified Method in the SNI Mouse Model

**Figure 6:**
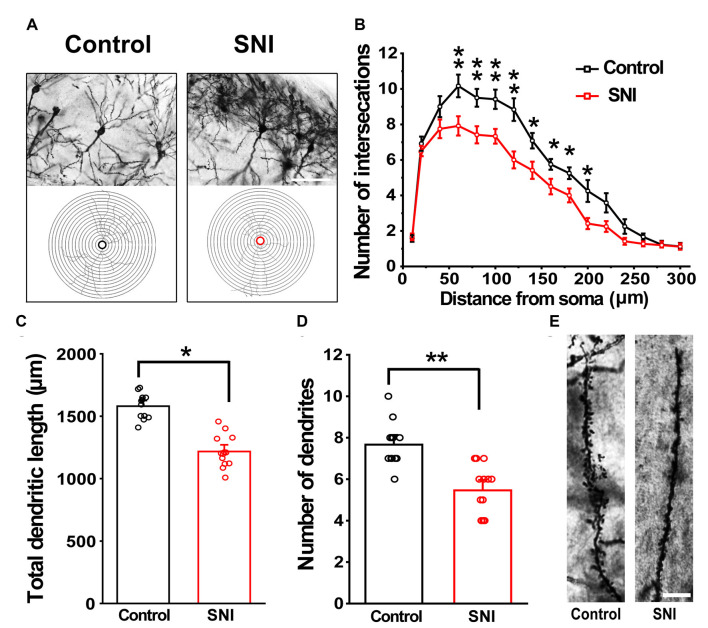
Modified Golgi-Cox staining showing the differences in hippocampal neurons between control and SNI mice. Schematic photomicrographs of neurons (20×) with the allocation of dendrites between repeated 20 μm-spaced concentric rings **(A)**. SNI mice showed a reduced number of neuronal intersections in the hippocampus compared to controls (at 100–240 μm from the soma) **(B)**. SNI mice showed a reduced total dendritic length and number of dendrites compared to control mice **(C,D)**. Pyramidal neurons of SNI and control mice were imaged under a 40× lens **(E)**. Scale bar, 100 μm. Each value represents mean ± SEM; **p < 0.01, *p < 0.05.

Hippocampus sections were selected for analysis. We traced each neuron using a camera lucida technique. A total of 12 neurons from six brain slices were analyzed per group (three animals in each group). Figure 6 shows the morphology of dendrites and axons in hippocampal neurons of the SNI and control mice. The stained neurons had clear cell bodies and well-defined dendritic spines in both SNI and control mice (Figure 6A). The number of intersections of dendrites and neurons in the SNI mouse hippocampus at 90–240 μm was significantly reduced compared with that in the control (*F*_(1,11)_ = 6595.149, *p* < 0.01; Figure 6B). Similarly, the SNI mice showed decreased dendritic length (*F*_(1,24)_ = 125.683, *p* < 0.05; Figure 6C) and number of dendrites (*F*_(1,24)_ = 22.149, *p* < 0.01; Figure 6D). These results are consistent with the notion that SNI mice exhibit damaged neuronal morphology in the hippocampus (Cichon et al., 2018).

## Discussion

In this study, we present a modified Golgi-Cox staining protocol that is simple and convenient, and yields improved results. Golgi-Cox staining is a powerful technique for the study of neuronal and glial morphology (Kartalou et al., 2020; Narayanan et al., 2020). However, its major drawbacks are that it is time-consuming, requiring at least 2 weeks for impregnation (Chu, 2006; Friedland et al., 2006), and it can result in brain slices fragmenting and detaching from glass slides (Chu, 2006). Although there have been attempts to improve Golgi-Cox staining (Table 1), the results have not been ideal. Our improved Golgi-Cox protocol produces high-quality staining results within a short time.

**Table 1:** Comparison of the impregnation of Golgi stain into neurons under various conditions used by different methods.

Tissue sample	Animals	Pre-processing	Impregnation time (days)	Impregnation temp (°C)	Methods overview	References
Neurons	Adult rats	ICP with 4% PFA	1	37	Tissue blocks were maintained at 37 ± 1°C during chromatin for only 24 h.	Ranjan and Mallick (2010)
Glial cells	Adult rats	No role of ICP in specific glial staining	2	37	Exposure (at any stage) the brain to a fixative 4% PFA increased the number of stained glia.	Ranjan and Mallick (2012)
Embryonic neurons	Mouse	ICP with 4% PFA followed by post-fixation with 8% GA in 4% PFA	14	RT	Introducing an additional aldehyde fixation step before impregnation.	Koyama and Tohyama (2012)
Primary hippocampal neurons	Rats	ICP with 4% PFA and 12.5% GA	2	RT	(1) Neurons fixed with a mixture containing 4% PFA and 12.5% GA before impregnation; (2) Rapid freezing of the fixed neurons using dry ice; (3) Immersion of the fixed neurons in antibody for visualization.	Koyama et al. (2013)
Dorsal hippocampus and Amygdala neurons	Adult rats	-	7	RT	Different impregnation duration effects on optimized staining of the dorsal hippocampus and basolateral amygdala were investigated.	Narayanan et al. (2014)
Glial cells	Adult rats	ICP with 4% PFA	16	26	Golgi-Cox staining was Observed at different temperatures (26°C and 37°C) during Golgi staining and fixed with PFA.	Gull et al. (2015)
Striatum neurons	Mouse	ICP with 4% PFA	14	RT	Gives high-quality staining on brain tissue blocks perfusion-fixed with 4% PFA and post-fixed by immersion for 24 h.	Bayram-Weston et al. (2016)
Hepatic stellate cells	Rats	-	15	RT	The tissues were stored in the dark for 15 days in 20 mL of Golgi-Cox solution and sectioned, 200 μm thick, immersed in a 15% sucrose solution using a vibratome.	Gómez Villalobos et al. (2017)
Cortex and Hippocampal neurons	Adult rats	-	2	RT	This method is optimized for using clarity and cubic that can be used in both fresh and fixed tissue.	Kassem et al. (2018)

*Abbreviations: ICP, intracardial perfusion; PFA, paraformaldehyde; GA, glutaraldehyde; RT, room temperature*.

First, we incubated tissue in fixative for 10, 40, or 70 min and evaluated the effect of fixation time on staining. We found that the best staining quality was achieved when brains were incubated in 4% PFA for 40–70 min; 10-min incubation resulted in high background and incompletely stained cell structure, whereas 40- and 70-min incubation produced images showing complete cell structures with sharp dendritic spines.

Next, we incubated brain tissues in the Golgi-Cox staining solution for four different periods (i.e., 36, 48, 72, and 144 h) to determine whether the overall time of the staining procedure can be reduced using our modified approach. In the traditional method, brain tissues are kept in dark at RT and the staining solution needs to be replaced every 48 h for approximately 10 16 days (Chu, 2006; Friedland et al., 2006). In our modified protocol, brain tissue incubated in staining solution for 48 h at 37°C showed improved staining of pyramidal neurons. However, 144-h incubations caused the disappearance of many neurons, indicating that the shorter incubation time of 48 h should not be exceeded. Thus, the modified method reduces the impregnation period by 36–48 h and overall experimental time by 8 days.

The other major problem with the traditional method, that long-term preservation at 4°C causes fragmentation of brain slices, was addressed by preserving brain slices at 4, −20, and −80°C, followed by Golgi-Cox staining. This showed that preservation at −80°C reduced fragmentation. We then compared the traditional protocol (26 days) with our modified protocol (8 days) using SNI and control mice. The modified protocol resulted in images with complete dendrite morphology against a clear background, similar to the traditional method but within a shorter experimental time. Furthermore, the improved protocol overcomes the shortcomings of slice fragility usually associated with the traditional method. It also promotes uniform crystallization and increases the reliability and reproducibility of neuronal staining.

The following points need to be considered when performing the proposed protocol: (1) residual blood on brain slices can interfere with the staining, so blood should be carefully washed out by perfusing mice with saline; (2) small immersion volumes of staining solution could lead to a decrease in the sensitivity and reliability of Golgi-Cox staining; thus, at least 5 ml staining solution is needed for every cm^3^ of brain tissue Kassem et al. (2018); (3) Golgi-Cox staining solution is sensitive to light, hence brown bottle must be used for incubation and keep it at dark; (4) to observe complete dendritic spines, the thickness of the brain slices should be at least 80 μm; and (5) using coated slides for staining can prevent brain slices from detaching during the staining process (Gómez-Villalobos et al., 2009).

To validate our modified method, we used SNI mice—a model for neuropathic pain (Richner et al., 2011). Previous reports associate this model with altered neuronal plasticity and pain-related protein expression in the hippocampus, as well as morphological changes (Metz et al., 2009; Tyrtyshnaia and Manzhulo, 2020). Our results are consistent with that of previous reports that SNI mice exhibit a significant reduction in the number of intersections, total dendritic length, and the number of dendrites. Our modified Golgi–Cox method optimized the process and enabled stable impregnation of neurons and visualization of complete neurons, including cell bodies, full-length dendritic trees, and even clear spine morphology.

Taken together, our modified Golgi-Cox staining method is cost-effective, fast, and reliable; it overcomes the shortcomings of the traditional method in terms of overall experimental time, slice fragility, and fragmentation of brain slices after long-term preservation. Furthermore, it promotes uniform crystallization and can easily be executed in most laboratories for the study of neuronal morphology in various brain regions.

## Data Availability Statement

The original contributions presented in the study are included in the article/Supplementary Materials, further inquiries can be directed to the corresponding author.

## Ethics Statement

The animal study was reviewed and approved and all experiments and animal housing were carried out following procedures approved by the Ethics Committee for animal research at South China Normal University, consistent with the guidelines for the care and use of laboratory animals established by the National Institutes of Health.

## Author Contributions

J-wZ designed the experiment, conducted Golgi-Cox staining, statistical analyses, and assisted in drafting the manuscript. ST wrote the manuscript and generated the figures. J-xJ established the experimental animal model. CL supervised the study and conducted a critical revision of the manuscript. All authors contributed to the article and approved the submitted version.

## Conflict of Interest

The authors declare that the research was conducted in the absence of any commercial or financial relationships that could be construed as a potential conflict of interest.

## Supplementary Material

SUPPLEMENTARY FIGURE 1 Comparison of 24 h and 36 h impregnation of brain tissues in an optimized Golgi-Cox staining solution.

SUPPLEMENTARY TABLE 1 Comparison of the impregnation of Golgi-Cox stain into neurons under various conditions according to various methods.
