# Supplementary figures and images for: Optimized Golgi-Cox Staining Validated in the Hippocampus of Spared Nerve Injury Mouse Model
Source: Front Neuroanat. 2020 Nov 9;14:585513. doi: 10.3389/fnana.2020.585513 (PMC7680754; doi:10.3389/fnana.2020.585513)

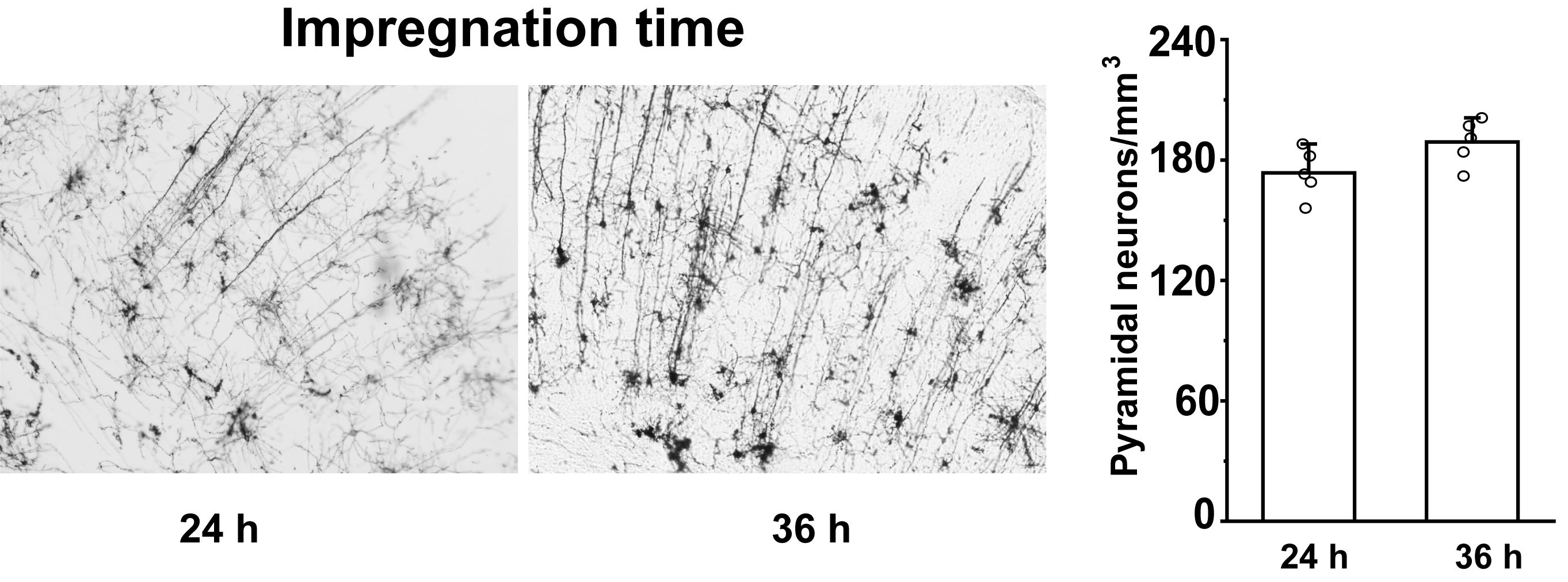

Supplement: SUPPLEMENTARY FIGURE 1 — Comparison of 24 h and 36 h impregnation of brain tissues in an optimized Golgi-Cox staining solution. [file Image_1.TIF]
