# Supplementary material for: Optimized Golgi-Cox Staining Validated in the Hippocampus of Spared Nerve Injury Mouse Model
Source: Front Neuroanat. 2020 Nov 9;14:585513. doi: 10.3389/fnana.2020.585513 (PMC7680754; doi:10.3389/fnana.2020.585513)
Supplement: SUPPLEMENTARY TABLE 1 — Comparison of the impregnation of Golgi-Cox stain into neurons under various conditions according to various methods. [file Data_Sheet_1.PDF]

Table 1. Comparison of the impregnation of Golgi-Cox stain into neurons under various conditions according to various methods

Tissue sample	Animals	Pre-processing	Impregnation time (days)	Impregnation temp (°C)	Methods overview	References
Neurons	Adult rats	ICP with 4% PFA	2	26	Tissue blocks were maintained at 37 ± 1°C during chromatin for only 24 h	Ranjan A et al., 2010
Glial cells	Adult rats	No role of ICP in specific glia staining	2	37	Exposure (at any stage) of the brain to a fixative 4% PFA increased the number of stained glia	Ranjan A et al., 2012
Embryonic neurons	Mouse	ICP with 4% PFA followed by post-fixation with 8% GA in 4% PFA	14	RT	Introducing an additional aldehyde fixation step before impregnation.	Koyama Y et al., 2012
Primary hippocampal neurons	Rats	ICP with 4% PFA and 12.5% GA	2	RT	(1) Neurons fixed with a mixture containing 4% PFA and 12.5% GA before impregnation; (2) Rapid freezing of the fixed neurons using dry ice; (3) Immersion of the fixed neurons in antibody for visualization	Koyama Y et al., 2013
Dorsal hippocampus and amygdala neurons	Adult rats	-	7	RT	Different impregnation duration effects on optimized staining of dorsal hippocampus and basolateral amygdala were investigated.	Narayanan S N et al., 2014
Glial cells	Adult rats	ICP with 4% PFA	16	26	Golgi-Cox staining were Observed at different temperatures (26 °C and 37 °C) during Golgi staining and fixed with paraformaldehyde.	Gull S et al., 2015
Striatum neurons	Mouse	ICP with 4% PFA	14	RT	Gives high quality staining on brain tissue blocks perfusion-fixed with 4% PFA and post-fixed by immersion for 24 h.	Bayram-Weston Z et al., 2016
Hepatic stellate cells	Rats	-	15	RT	The tissues were stored in the dark for 15 days in 20 mL of Golgi-Cox solution and sectioned, 200 μm thick, immersed in 15% sucrose solution using a vibratome.	Gómez Villalobos M J et al., 2016
Cortex and Hippocampus neurons	Adult rats	-	2	RT	This method optimized for using clarity and cubic that can be used in both fresh and fixed tissue.	Kassem M S et al., 2018

Abbreviations: ICP, intracardial perfusion; PFA, paraformaldehyde; GA, glutaraldehyde; RT, room temperature.
